# Mathematical modelling and evaluation of the different routes of transmission of lumpy skin disease virus

**DOI:** 10.1186/1297-9716-43-1

**Published:** 2012-01-11

**Authors:** Reuma Magori-Cohen, Yoram Louzoun, Yael Herziger, Eldad Oron, Alon Arazi, Eeva Tuppurainen, Nahum Y Shpigel, Eyal Klement

**Affiliations:** 1 Department of Mathematics and Gonda Brain Research Center, Bar Ilan University, Ramat Gan, Israel; 2 Koret School of Veterinary Medicine, Robert H. Smith Faculty of Agriculture, Food and Environment, the Hebrew University, Israel; 3 Hachaklait, Mutual Society for Veterinary Services, Caesarea, Israel; 4 S.A.E. Afikim, Afikim, Israel; 5 Institute for Animal Health, Ash Road, Pirbright, UK

## Abstract

Lumpy skin disease (LSD) is a severe viral disease of cattle. Circumstantial evidence suggests that the virus is transmitted mechanically by blood-feeding arthropods. We compared the importance of transmission via direct and indirect contact in field conditions by using mathematical tools. We analyzed a dataset collected during the LSD outbreak in 2006 in a large dairy herd, which included ten separated cattle groups. Outbreak dynamics and risk factors for LSD were assessed by a transmission model. Transmission by three contact modes was modelled; indirect contact between the groups within a herd, direct contact or contact via common drinking water within the groups and transmission by contact during milking procedure. Indirect transmission was the only parameter that could solely explain the entire outbreak dynamics and was estimated to have an overall effect that was over 5 times larger than all other possible routes of transmission, combined. The *R*_0 _value induced by indirect transmission per the presence of an infectious cow for 1 day in the herd was 15.7, while the *R*_0 _induced by direct transmission was 0.36. Sensitivity analysis showed that this result is robust to a wide range of assumptions regarding mean and standard deviation of incubation period and regarding the existence of sub-clinically infected cattle. These results indicate that LSD virus spread within the affected herd could hardly be attributed to direct contact between cattle or contact through the milking procedure. It is therefore concluded that transmission mostly occurs by indirect contact, probably by flying, blood-sucking insects. This has important implications for control of LSD.

## Introduction

Lumpy skin disease (LSD) is caused by Lumpy skin disease virus (LSDV), a DNA virus of the family *Poxviridea *and of the genus *Capripoxvirus*. It is closely related and has high antigenic resemblance to sheep pox and goat pox viruses [1]. The disease is characterized by disseminated appearance of skin lesions, 2-5 cm in diameter and lymphadenopathy, accompanied by high fever which can sometimes exceed 41°C and may last up to 2 weeks [2]. Morbidity rate varies widely depending on the abundance of insect vectors and susceptibility of hosts ranging from 3 to 85% [3]. In general, mortality rate is low (1-3%) but in some occasions up to 75% mortality has been reported [1]. LSD is associated with significant production losses. It is therefore defined as a notifiable disease by the World Organization for Animal Health (OIE).

It is well accepted that lumpy skin disease is mechanically transmitted by different types of biting and blood-feeding arthropods, although the importance of the vectors in the transmission of the virus in field conditions is not fully understood. *Aedes aegypti *was found to successfully transmit the virus between cattle up to 6 days after feeding upon infected animals [4]. However, *A. aegypti *is absent from Israel, where 3 LSD outbreaks have been documented to date [5]. *Stomoxys calcitrans, Culicoides nubeculosus*, *Culex quinquefasciatus *and *Anopheles stephensi *failed to transmit the virus to susceptible cattle, although no transmission attempt was made immediately after feeding on infected cattle [6]. Since LSDV is transmitted mechanically, such a time frame for transmission is relevant, primarily with interrupted feeders like *Stomoxys calcitrans*. In light of its high abundance near previous outbreaks of LSD [7], this fly cannot be precluded yet as a potential mechanical vector for LSDV. Further studies are required to investigate the role of biting flies in the transmission of LSDV in experimental and field conditions.

In a study performed by Carn and Kitching, intradermal inoculation of LSDV in cattle caused generalized disease in less than 20% of cases while all other infected animals developed only localized disease. As opposed to this, over 70% of cattle infected intravenously showed generalized disease. Infection through the conjuctival sac was unsuccessful though it was attempted only on 2 animals. Individuals that lived for one month in close contact with the infected animals did not develop disease [8]. In another study, experimental infection showed low levels of virus in oral and nasal secretions 12 to 18 days post infection (dpi) [9]. Sharing of water troughs was also raised as a possible route for LSDV transmission [10].

The assumption regarding the high relative significance of lumpy skin disease transmission by arthropods is based either on data collected during experimental infection with the Neethling strain [8] or on circumstantial evidence of high abundance of blood-feeding arthropods near LSD outbreaks [7,11]. To date, there is no accurate data collected during natural outbreaks that can support these previous findings. It is thus not clear what is the relative significance of each of the suggested routes in such natural settings. In the current study we used mathematical modelling to understand the relative role of each transmission route (indirect contact, direct contact and transmission during the milking procedure). This was performed by analyzing data collected during the investigation and follow-up of an outbreak of lumpy skin disease in a large dairy cattle herd in Israel in 2006. Segregation of the groups in the herd prevented contact between most of them. We used a back-calculation method which was previously used for forecasting incidence of Human Immunodeficiency Virus [12] and Bovine Spongiform Encephalopathy [13]. This method uses incidence data, together with an estimate of the incubation period to glean information about past infection rates. By doing it for each cow and by knowing the actual incidence for every group we could estimate the probability of infection by each of the assessed transmission modes.

## Material and methods

### Study population

**Figure 1:**
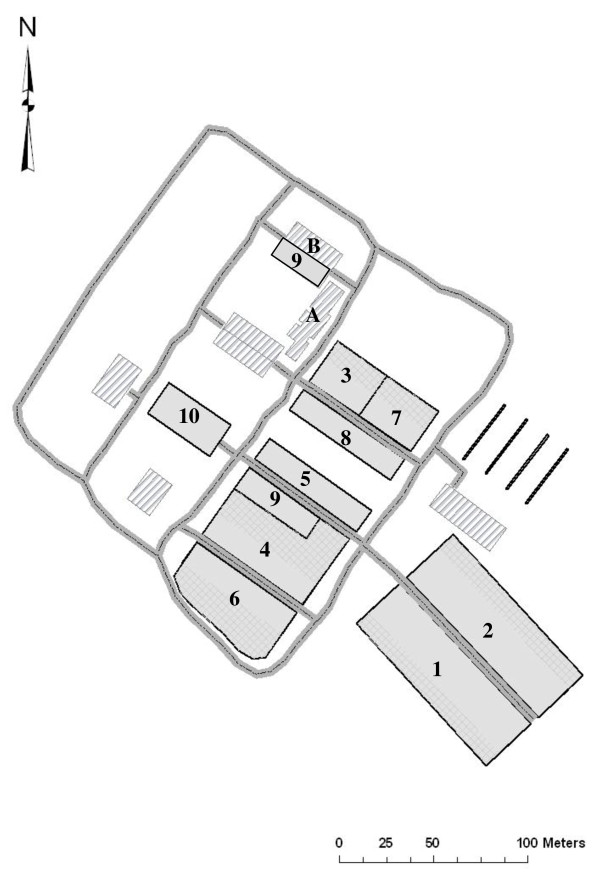
**Location of the groups in the affected herd**. A. Milking pallor B. Euthanasia area. 1. First lactation cows 2. Second lactation cows. 3. Pre-calving group 4. Replacement heifers pending insemination and in early pregnancy 5. Replacement heifers (10-12 months old) 6. Pregnant replacement heifers 7. Dry cows. 8. High parity 9. Fattening calves. 10. Suckling calves.

An outbreak of LSD was suspected in the dairy herd of Ein-Zurim on June 19^th ^after the appearance of typical lesions in a few cows. The herd is located in south western Israel. The outbreak occurred in a non-grazing intensive 610 head dairy farm which included 10 groups (Figure 1). The groups were separated from each other and did not share water troughs or feed bank. Water was provided separately to each trough by a central water system and each trough was drained separately. Three groups included lactating cows (groups 1, 2 and 8, *n *= 246), while the 7 other groups included either heifers in various stages (groups 4-6), cows at pre-calving stage (group 3), dry cows (group 7), fattening calves (group 9 which, as noted in Figure 1, was scattered in two cow sheds) or suckling calves (group 10). All groups were completely separated from each other except groups 9 and 4 and groups 3 and 7, which had common fences but did not have common water troughs or feed banks.

**Figure 2:**
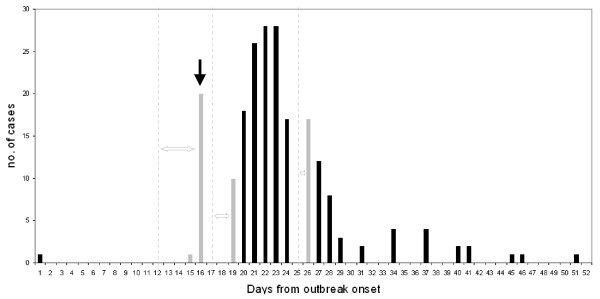
**Epidemic curve of generalized LSD during the outbreak in Ein-Zurim, 2006**. Black columns represent cases for which the date of disease appearance is definite. Grey columns represent cases for which date of disease appearance is not definite. The dashed lines represent their earliest assumed date of appearance. Double headed arrows represent the time period during which these cases may have first appeared. Black arrow represents vaccination date.

Epidemiologic investigation revealed that from May 15^th ^to May 25^th ^there was a cluster of 16 late pregnancy abortions that preceded the outbreak. Fourteen of the cows that aborted were pregnant heifers. All were moved to the group of cows in first lactation (group 1 Figure 1). It appeared that on June 7^th ^one of the aborting heifers was culled as a result of poor performance and body condition. The herdswoman reported that while placing this calf on the truck she noticed the abundance of numerous lumps scattered all over the calf's trunk and limbs. This calf is thus assumed to be the index case and this date is defined as day 1 of the outbreak. On June 22^nd ^(outbreak day 16), 21 cows and calves were culled after showing generalized lumpy skin disease. The exact date of onset of clinical signs in these cows and calves was not documented. However, after interviewing the veterinarian and the herdswoman, it was understood that generalized clinical signs of LSD were not apparent before June 18^th ^(outbreak day 12). Thereafter, the disease spread rapidly within the herd. Eventually, 206 animals were affected and culled with generalized LSD, leaving 39 with only localized lesions remaining in the herd. The last case appeared on July 21^st^. Thus, the total outbreak duration from the appearance of the first suspected case until the occurrence of the last case was 45 days (Figure 2).

### Data collection and intervention

**Figure 3:**
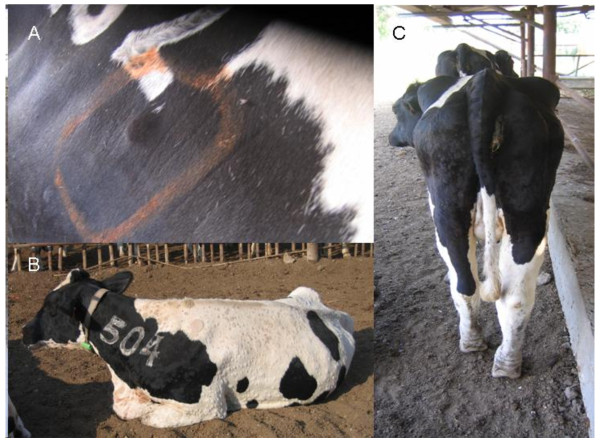
**Clinical appearance of lumpy skin disease during the outbreak in Ein-Zurim in 2006**. **A**. Localized lumpy skin disease. **B**. Generalized disease **C**. Generalized disease with limb oedema.

Starting from June 22^nd^, the whole herd was examined daily by a single clinician to detect cattle that showed typical LSD clinical signs. Suspected cattle were categorized as having either localized disease (i.e. no more than 3 localized lumps which were characterized by an induration of a diameter not higher than 5 cm) or a generalized disease, which was characterized by the appearance of multiple indurations all over the trunk, limbs and face (Figure 3), and was sometimes accompanied by high fever, lymphadenopathy, limb oedema and reduction in milk production. Cattle that showed suspected lesions were examined again the next day to monitor for the development of generalized clinical signs. Cattle were culled during the same morning in which generalized disease was detected, while those that showed only small confined lesions were left in the herd. On June 22^nd ^the whole herd was vaccinated with a live-attenuated sheep-pox vaccine (strain RM-65 (Abik^®^, Israel)) (Figure 2). Groups were vaccinated by separate needles, but in some of the cases cattle from the same group were vaccinated by the same needle.

### Laboratory diagnosis

Lumpy skin disease virus was identified in seven cows by a gel-based polymerase chain reaction (PCR) [14]. Virus isolation and electron microscopy were performed on excised skin lesions as described by Brenner et al. [15]. Following virus identification in the first cases, diagnosis was mostly performed on the basis of clinical signs according to the case definition described in the previous section.

### Dynamic transmission model

For the outbreak analysis, cases of LSD were defined as those having generalized clinical disease. The following assumptions were taken to explain the outbreak dynamics:

• Except for the first few days, sick cattle were removed when detected in the morning. Thus, each cow was infective for only a few hours.

• All cows are susceptible to infection by LSD and development of generalized morbidity. Since there is no evidence in the literature to support this assumption, its influence on model results was further tested by sensitivity analysis.

• The disease has an incubation period, with a given distribution *f*(Δ*t*), whereΔ*t *is the time interval from infection to the appearance of clinical signs. Cattle are infective only on the appearance of clinical signs (i.e. incubation period equals the latent period).

• Cattle can infect other cattle via four possible routes A) long distance transmission between the groups, possibly by blood-sucking flying arthropods with a rate of *alpha*. Since the model describes transmission within the farm, in which distance does not exceed 300 meters, this parameter was not defined as distance dependent. Therefore, the probability of the transmission of virus by this route from an infectious cow to a susceptible one in the same group equals the probability of virus transmission from the same cow to a cow in another group. B) Infection by direct contact, not during milking, with a rate of *beta*. Since there is no contact between the cows from different groups (apart from the contact during milking), this type of transmission is possible only within each group. **C) **Transmission of virus during the milking procedure, with a rate of *gamma*. As all relevant groups were milked at the same milking pallor (A, Figure 1) and no measures were put in place to prevent transmission, such transmission could occur within the group or between groups of lactating cows (i.e. groups 1, 2 and 8), and D) Transmission between bordering groups (i.e. groups 4 and 9 and groups 3 and 7) with a rate of *delta*.

• 21 sick cattle heads were detected during the few days prior to day 16, while no sick cattle were observed at day 11. We thus assumed a cumulative growth in the number of infected cows between day 11 and day 16. Two different case accumulation patterns were tested, as shall be further explained.

• The cattle that showed clinical signs before day 16 (early cases) stayed for a long time in the herd, and were culled only after a few days, while cattle that became sick later stayed in the herd only for a few hours until culling. Therefore, the early cases had more time to infect other cattle than the late cases. Thus, for the early period until day 16, probability of infection is related to the prevalent cases while for the late period it is related to the number of new cases appearing each day. Furthermore, since the early cases remained for the whole day in the herd while the late cases stayed for only a few hours until culling, we assume that the infectivity of the late cases is smaller by a constant *c *than earlier cases.

These assumptions were combined to produce a model in order to explain the outbreak dynamics. We define a probability of *Pij *that a cow in group *j *at day *i *would express clinical signs, given that it has not yet done so. The total probability *Pij *for showing clinical signs can thus be defined as:

(1) $$P_{ij}=\alpha \underset{k\in \text{all}\;\text{groups}}{\sum }x_{ik}+\beta x_{ij}+\gamma \underset{k\in \text{join}\;\text{miliking}\;\text{groups}}{\sum }x_{ij}+\delta \underset{k\in \text{neighboring}\;\text{groups}}{\sum }x_{ik}$$

where *x_ij _*and *x_ik _*are the approximated numbers of infectious cows in the respective groups in the respective days as computed through a convolution with the appropriate incubation period (i.e. their sums multiplied by the transmission parameters represent the sums of previous prevalences, weighted by their contribution to clinical incidence at day i). The first term includes the total number of the infectious cows in the herd (including the cows in the same group). The second term includes only the cattle from the same group of the animals for which the probability of infection is calculated. The third term includes only lactating cows *in different groups *(groups 1, 2 and 8) given that the susceptible cow is lactating, and the last term includes only cattle from the groups bordering with groups 3, 4, 7 and 9, (not including the self-group). The analysis was performed on the 10 groups for each of the 50 days of surveillance.

Since only the onset of clinical signs can be detected we cannot determine the precise time of infection of each cow. Therefore, we used a weighted combination of the number of infected cows in previous days to compute the infection probability. Formally, each *x_ij _*is computed as a convolution of the number of infectious cows in each of the days preceding day i with a Gaussian distribution representing the distribution of incubation periods between exposure to the virus and clinical (and infectious) state: The following convolution formula was used for *x_ij_*:

(2) $$x_{ij}=\underset{i^{*}<i,j}{\sum }\frac{1}{\sqrt{2\pi \sigma }}e^{-\frac{{\left(i-i^{*}-\mu \right)}^{2}}{2^{*}\sigma^{2}}}y_{i^{*}j}$$

where *y*_i*j _is the number of sick cows in group j* at day i*, *μ *is the estimated incubation period and *σ *is the standard deviation around this estimate.

In order to use realistic figures we searched the literature for experimental studies in which exact incubation period from infection to generalized disease was reported. Three such studies were found [8,9,16]. The average incubation period reported in these studies was 7 days for virus isolation from blood and 11 days for generalized disease (Table 1). We therefore fitted the average incubation period for the model, limiting its value to the range of 7 to 11 days. The standard deviation for the incubation period was fitted as well.

**Table 1:** Incubation period in 3 LSD clinical infection studies

Reference	Titer (TCID_50_)	Measured outcome		Number of animals showing outcome in each dpi															
				**1**	**2**	**3**	**4**	**5**	**6**	**7**	**8**	**9**	**10**	**11**	**12**	**13**	**14**	**Average**	**SD**

[16] (Tuppurainen *et al.*)	10^5.4^	Generalized disease	IV						1	1								6.5	0.7
		Virus isolation *							2									6	0

[8] (Carn & Kitching)	10^2^-10^6.5^	Generalized disease	ID							1						1	2	12	3.4
		Virus isolation				1												3	NA
		Generalized disease	IV									4			2	2		10.75	1.9
		Virus isolation							1			2						8	1.7

[9] (Babiuk *et al.*)	10^5.25^	Generalized disease	IV						4*									NA	NA
		Virus isolation							2			2						7.5	1.7

**Total**		Generalized disease***																**10.5**	**2.8**
		**Virus isolation**																**6.9**	**2**

* Isolation of virus from blood.

** Generalized disease is reported to occur between 6 to 10 dpi. Exact onset of clinical signs was not reported.

*** Without [9].

As previously mentioned, the exact date of onset of LSD clinical signs was not determined for the first 21 cows/calves. We thus reconstructed the outbreak using two scenarios, with different accumulation during the first 5 days preceding outbreak day 16; either a cumulative linear increase in the number of prevalent cattle showing clinical signs (i.e. constant number of incident cases and a respective rounded number of prevalent cases for days 11-16: 0, 4, 8, 12, 17, 21) or a cumulative exponential increase in the prevalent number of infected cattle showing clinical signs (i.e. linear increase in number of incident cases and rounded number of prevalent cases for days 11-16: 0, 0, 1, 5, 12, 21). The constraint for all of these scenarios was that on outbreak day 11 no cases existed in the herd and on outbreak day 16 there were 21 generalized LSD cases in the herd which were culled in the same day.

The optimization analysis for both models (cumulative and exponential increase in the prevalent number of infected cattle showing clinical signs) was performed simultaneously on seven parameters: *alpha, beta, gamma, delta *- the transmission parameters, *mu *and *sigma *- the average and the standard deviation in the incubation time and *c *the ratio between the infectivity after the disease was detected and the infectivity during the first few days.

### Estimation of model parameters and statistical analysis

Parameters were estimated by maximizing the fit to the observed (clinical) incidence in time. Assuming that each infected cow is an independent event that is not affected by other infected cows in the same herd on the same day, the number of sick cows should obey a binomial likelihood function:

(3) $$\underset{i}{\prod }\underset{j}{\prod }\left(\begin{array}{c} N_{ij}\\ y_{ij} \end{array}\right)p_{ij}^{y_{ij}}{\left(1-p_{ij}\right)}^{\left(N_{ij}-y_{ij}\right)}$$

We first performed an analysis which included the constant *c *which scales for the lower infectivity of later cases (during control), the incubation time parameters (*mu *and *sigma*) and each one of the transmission parameters separately (four parameters) to explore if the outbreak scenario could be repeated using each transmission parameter alone (i.e. first *mu*, *sigma*, *c *and *alpha*, then *mu*, *sigma*, *c *and *beta *and so on). The analysis was continued by including the transmission parameter that showed the most significant contribution to the model, and then by inserting the other parameters one by one and testing the statistical significance of their contribution by examining the change in the -2 log maximum likelihood, which is χ^2 ^distributed. We further tested the association of morbidity with cattle density (number of cows/m^2^), age group (cows vs. calves, which include suckling calves and fattening calves and all heifers before first calving or aborting heifers) and lactation status (*eta*, *teta *and *nu *in equations 4, 5 and 6, respectively). These risk factors were tested in a model which included *alpha*, *beta *and *gamma*. Lactation status was also tested in a model which included only *alpha *and *beta *since this risk factor overlaps with the definition of *gamma *(transmission during the milking procedure).

The following models were used:

(4) $$P_{ij}=\left[\alpha \underset{k\in \text{all}\;\text{groups}}{\sum }x_{ij}+\beta x_{ij}+\gamma \underset{k\in \text{join}\;\text{milking}\;\text{groups}}{\sum }x_{ik}\right]\left(1+\eta \frac{N_{ij}}{A_{j}}\right)$$

for examining the association of cow density with incidence, where *A_j _*is the total area of each cow shed.

(5) $$P_{ij}=\left[\alpha \underset{k\in \text{all}\;\text{groups}}{\sum }x_{ik}+\beta x_{ij}+\gamma \underset{k\in \text{join}\;\text{milking}\;\text{groups}}{\sum }x_{ik}\right]\left(1+\theta H_{j}\right)$$

for examining the association of cattle age (calf or cow) with incidence, where *H_j _*is an indicator variable, equals to 0 for young calves or 1 for adult cows.

(6) $$P_{ij}=\left[\alpha \underset{k\in \text{all}\;\text{groups}}{\sum }x_{ik}+\beta x_{ij}+\gamma \underset{k\in \text{join}\;\text{milking}\;\text{groups}}{\sum }x_{ik}\right]\left(1+v*mg_{i}\right)$$

for examining the association of lactation status with incidence, where *mg_i _*is an indicator variable, equals to 0 for cattle that were not milked during the outbreak or 1 for cows that were during the lactation period.

The optimal parameters were computed using a Nedler-Mead minimization algorithm [17] implemented on MATLAB^® ^software. The algorithm was initiated from multiple starting points to ensure a global minimization. The results were similar for most initial starting points.

### Sensitivity analysis

We performed a sensitivity analysis for each pair of parameters in order to analyze their influence on the maximum likelihood function of the model when compared to the real outbreak data.

Further sensitivity analyses were performed to test the robustness of model results to assumptions regarding mean and standard deviation of the incubation period and regarding the existence of subclinical infection. In the first analysis, we set these parameters to be constant and searched for the optimal values of *alpha, beta *and *gamma*, in order to test if the ratio (*beta + gamma*)/*alpha *remains small over a range of means and standard deviations of the incubation period.

The current model assumes all cows to be susceptible to infection by LSD and development of generalized morbidity. If this assumption is not correct the number of susceptible cattle is expected to be diluted by non-susceptible cows (which may be only sub-clinically infected). We therefore tested the effect of this assumption by assuming a varying number of susceptible cows (out of those that never showed clinical signs).

### An alternative model

The model studied here is the most straightforward model, where clinical incidence is proportional to the number of sick cows in the days before. An alternative model can be proposed where, for direct transmission, the clinical incidence is proportional to the proportion of sick cows in the days before. We tested the fit of this model by multiplying *beta *by x_ij_*[N]/N_ij _instead of x_ij _and *gamma *and *delta *(all associated with direct transmission) by the sum over x_ik_*[N]/N_ij_, instead of the sum over x_ik_, where [N] is the average number of cows over days and groups.

### Alternative fitting method

An alternative approach would be to use the clinical incidence data for reconstruction of infection incidence, using the incubation period distribution. Then, infection incidence can be fitted to the prevalence of infection. This alternative method, which qualitatively gives similar results, is described in the additional file 1.

## Results

### Analysis of transmission parameters

**Figure 4:**
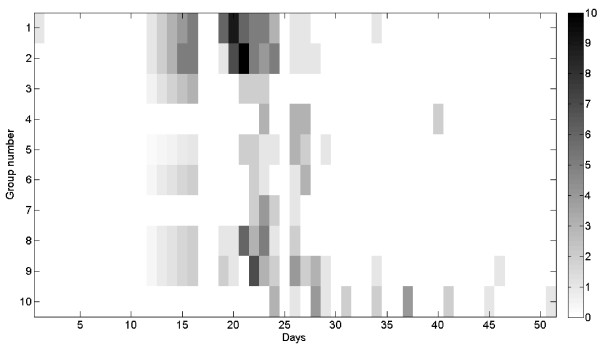
**Spread of generalized LSD between cattle groups**. See Table 2I for group description. Grey scale indicates daily incidence in each group.

A significant difference in incidence rate was observed between the groups, with the suckling calf group being the last group to be affected and showing the lowest incidence (20%) and the pre-calving group showing the highest incidence (56%) (Table 2). The analysis of outbreak time course revealed that initial spread of the disease from the index case (which was located in group 1) occurred simultaneously to 7 groups (groups number 1, 2, 3, 5, 6, 8, 9) in the herd. All of which did not have any direct contact with each other (except for contact during the milking procedure between groups 1, 2 and 8) and did not share water troughs or feed bank (Figure 4). Thus one can conclude that the observed outbreak cannot be solely explained by the direct contact mode of transmission.

**Table 2:** Univariate analysis of the risk for generalized LSD in the outbreak in Ein-Zurim, 2006

Group (group number)	Attack rate	Relative risk (95% CI)	*P *value*
First lactation cows (1)	43/105 (41%)	Reference	NA
Second lactation cows (2)	41/100 (41%)	1 (0.72-1.39)	1
Pre-calving (3)	9/16 (56.2%)	1.37 (0.84-2.24)	0.29
Replacement heifers pending insemination and in early pregnancy (4)	11/36 (30.6%)	0.75 (0.43-1.28)	0.32
Replacement heifers (10-12 months old) (5)	13/60 (21.7%)	0.53 (0.31-0.9)	0.016
Pregnant replacement heifers (6)	9/29 (31%)	0.76 (0.42-1.37)	0.39
Dry cows (7)	9/34 (26.5%)	0.65 (0.35-1.18)	0.16
High parity (8)	21/41 (51.2%)	1.25 (0.86-1.82)	0.27
Fattening calves (9)	29/84 (34.5%)	0.84 (0.58-1.22)	0.45
Suckling calves (10)	21/105 (20%)	0.49 (0.31-0.76)	0.002

*Chi-squared test

### Results of the dynamic model

In all cases, the optimal fit was achieved when the incubation period was 7 days with a standard deviation of approximately two days (Table 3). The model which assumed a cumulative linear increase in the number of prevalent cattle showing clinical signs during the early stage of the outbreak produced a better fit than the model which assumed a cumulative exponential increase in this number (Table 3). Therefore, cumulative increase of prevalence between days 11-16 was used for further analyses.

**Table 3:** Assessment of transmission parameters for the outbreak of LSD in Ein-Zurim in 2006^¥^

	*alpha* (Indirect contact)	*beta* (direct contact)	*gamma* (transmission between lactating groups)	*delta* (bordering groups)	*eta *(density)	*teta *(age)	*nu *(lactation status)	Constant (*c*)	*mu*	*sigma*	L*	p-value^€^
***alpha+beta+ gamma+delta***	0.026	0.006	0.011	0.001				0.2	7	1.741	816.31	0.65
***alpha+beta+ gamma***	0.026	0.006	0.011					0.2	7	1.724	816.51	0.03
***alpha+beta***	0.032	0.008						0.2	7	1.797	820.98	0.0004
***alpha+gamma***	0.028		0.011					0.27	7	1.008	827.32	0.015
***alpha**^§^*	0.038							0.22	7	1.231	833.22	
***alpha+beta+ gamma+eta *(Eq. 4)**	0.023	0.0116	0.0069		-0.0032			0.2	7	1.88	815.88	0.43
***alpha+beta+ gamma+teta *(Eq. 5)**	0.024	0.008	0.009			0.0013		0.2	7	1.79	815.82	0.41
***alpha+beta+ gamma+nu *(Eq. 6)**	0.024	0.0088	0.0079				0.0044	0.2	7	1.48	816.25	0.61
***alpha+beta+nu ***	0.023	0.0155					0.0002	0.2	7	1.85	817.24	0.053
***alpha+beta+ gamma+delta *linear increase in the number of incident cases until day 16**	0.031	0.014	0.008	0.000005				0.2	7	2.19	829.5	
***alpha+beta+ gamma+delta *(*beta+ gamma+delta *) are associated with incidence**	0.033	0.008	0.002	0.000014				0.2	7	1.81	824.58	

^¥ ^Unless stated otherwise, a constant incidence prior to day 16 was assumed and all transmission parameters were associated with the number of cases and not incidence.

* L = -2log (likelihood).

**€ ***p*-value for the addition of the last parameter.

§ The model without alpha has a likelihood of infinity.

**Figure 5:**
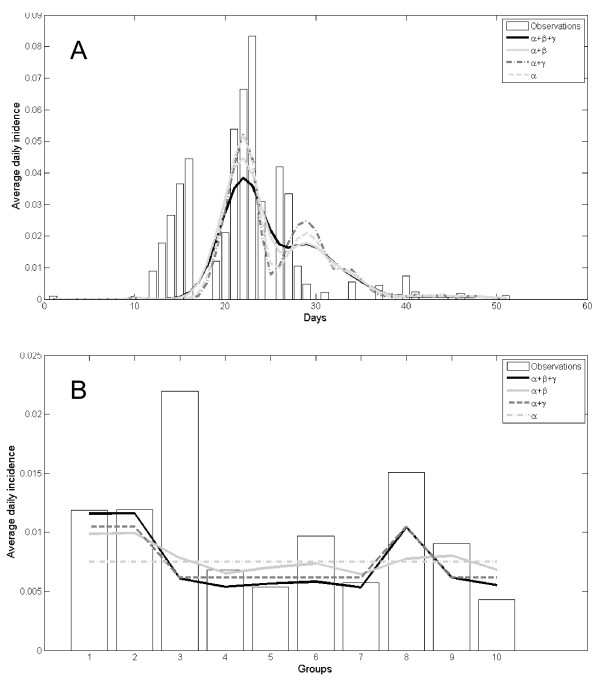
**Fit of transmission model with actual data of LSD outbreak in Ein-Zurim during 2006**. Bars - actual data. Each line represents a model. Note that the parameters of each model were optimized independently. **A**. Epidemic curve. **B**. Average daily incidence in each group during the outbreak period.

The outbreak scenario could be explained by a model that contains only *alpha *(i.e. same probability for transmission within and between cow sheds) (Table 3). However, the addition of *beta *or *gamma *(i.e. transmission only within the groups or during milking procedure) showed a statistically significant, yet minor increase in the model fit (Table 3, Figure 5).

**Figure 6:**
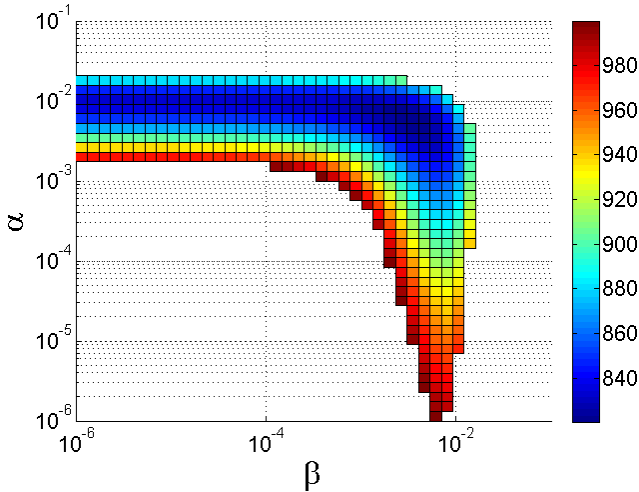
**Sensitivity analysis of alpha (indirect contact) and beta (direct contact)**. Surface color represents maximum likelihood for the model fit with the real data of LSD outbreak in Ein-Zurim, 2006. Note, that the model is significantly more sensitive to *alpha *than to *beta*, primarily around the optimal value.

Addition of *delta *to account for transmission between bordering groups did not show any statistically significant change of the maximum likelihood. As can be shown in Figure 6, sensitivity analysis revealed high sensitivity of the model to *alpha *as opposed to low sensitivity to *beta*, though best fit is achieved by inclusion of both parameters in the model. A similar result is obtained when *gamma *is introduced (data not shown). The point estimates of the best model indicate that the infection risk imposed by a cow by indirect transmission (*alpha*) is about five times larger than by direct transmission (*beta*), and more than two times larger than the contribution of transmission by milking (*gamma*) (Table 3, line 1). Because the number of cows that are interconnected by indirect transmission is approximately ten times larger than the number of cows that are linked by direct contact the total contribution of indirect transmission is 20-50 times larger than the contribution of direct transmission. The contribution of each transmission modes to the basic reproductive number (*R*_0_) during the initial stage of the outbreak can be calculated per 1 day of abundance of an infectious cow in the herd. Given a total number of approximately 600 cows, *R*_0 _per one day for indirect transmission alone (*alpha*) is 0.026 × 600 = 15.6 and *R*_0 _for direct transmission alone (*beta*) is 0.006 × 60 = 0.36. *R*_0 _for transmission through milking procedure (gamma) is 0.011 × 246 = 2.7.

The addition of cow density, lactation status and age to the full dynamic model (which included *alpha*, *beta *and *gamma*) did not significantly increase the fit of the model with the observed data (Table 3). However, when lactation status was added to a model which included only *alpha *and *beta *its contribution was close to statistical significance (*p *= 0.053).

### Sensitivity analysis for the mean and standard deviation of the incubation period

**Figure 7:**
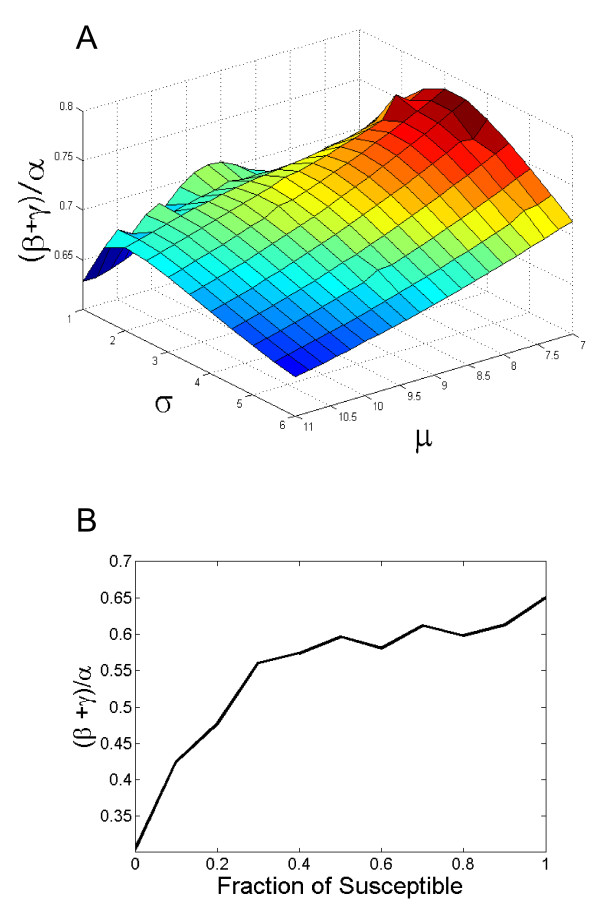
**Ratio between direct (beta + gamma) and indirect (alpha) transmission rates as a function of**: **A**. Fixed average and standard deviation of the incubation time. **B**. Ratio of susceptible cattle out of cattle that did not show clinical signs.

In order to test that the results of this model are not sensitive to the precise details of the incubation period, we repeated the analysis with fixed incubation period average time and standard deviation, and looked for the optimal values of *alpha*, *beta *and *gamma*. In all values used for incubation period the ratio *(beta + gamma)/alpha *was significantly lower than 1 (Figure 7a). It should be emphasized again that *alpha *is then multiplied by a much larger number of cows than *beta*. Thus the effective ratio is 5-10 times smaller than observed in the sensitivity analysis.

Another sensitivity analysis tested the influence of the assumption that all cattle are susceptible to generalized clinical infection by LSDV. While reducing the number of susceptible cows had an effect on the total force of infection computed, it only further reduced the ratio *(beta+gamma)/alpha *(Figure 7b).

### Alternative model

The model in which direct transmission was associated with incidence of infectious cattle performed worse than the model in which all transmission parameters were associated with the absolute number of infectious cattle (Table 3). In this model the ratio *(beta + gamma/alpha *was 0.32.

### Alternative fitting method

The alternative fitting method, linking back calculated infection incidence to prevalence, gave similar results producing an even smaller ratio between direct and indirect transmission (See additional file 1, Figure S1).

## Discussion

The results of this study indicate that in the outbreak described, transmission of LSDV by direct contact had only a minor effect on virus spread. These findings were found to be very robust to various assumptions regarding mean and standard deviation of incubation period and regarding the existence of subclinical infection.

Low significance of direct transmission as opposed to the high relative importance of indirect transmission coincides with the outcome of previous experimental studies [8,9] and was supported by several findings from this study. The initial spread of the disease from the index case occurred simultaneously to 7 groups in the herd that had not been in any direct contact with each other. This suggests that the virus must have been transmitted by indirect routes. The insignificance of the transmission by direct contact may also be supported by the fact that in two of the later affected groups (groups 4 and 7), only 3 to 5 days passed from onset of clinical signs in initial cases to appearance of clinical signs in at least 90% of the affected animals. Since at this stage active surveillance was already conducted, there is a precise documentation of onset of clinical signs in all cases. This time period, which is about half of the assumed average incubation period of LSD (which averages between 7 to 11 days as described in Table 1), shows that secondary transmission within these groups hardly took place. Therefore, most of the cases in groups 4 and 7 must have occurred as a result of indirect transmission of the virus from other groups.

The conclusion is further supported by the transmission model which indicates that the outbreak could be explained almost entirely by inclusion of one parameter (*alpha*) which represents transmission by indirect contact within and between groups. Inclusion of another parameter for transmission within the group does significantly increase the model fit with the observed data. However, its actual effect is low (Figure 5) and sensitivity analysis shows that its influence on the maximum likelihood of the model results is minor (Figure 6). Furthermore, this increase can also be explained by distance, e.g. because of an increased probability of mechanical transmission by flying biting insects, between adjacent cows.

From a practical point of view, the results from this study imply that the contribution of indirect transmission to *R*_0 _is significantly higher than that of direct transmission. *R*_0_, attributed to *gamma *(transmission between lactating groups), was 2.7. Though it was previously suggested that LSD can be transmitted by milk [10], the interpretation that transmission during milking procedure had a significant contribution to the spread of this outbreak should be taken very cautiously. First, *gamma *is only marginally significant (Table 2; *p *= 0.03). Second, since the two largest lactating groups are located very close to each other, *gamma*, like *beta *can actually represent higher probability of indirect mechanical transmission to individuals that are in high proximity to infectious cattle. Third, *gamma *can be influenced by higher susceptibility of lactating cows to generalized disease [18]. Indeed, when instead of *gamma *we added a parameter to account for higher susceptibility of lactating cows to clinical infection (*nu*, Table 3), its contribution to model fit was close to statistical significance and was very close to the contribution of *gamma*. In any case, it should be remembered that *gamma *is relevant only for 40% of the cattle in this herd and for only 51% of the affected cattle, which were scattered in three out of the 10 groups involved in this outbreak. Thus, while indirect transmission can explain the disease spread, it cannot be explained by direct transmission or by transmission during the milking procedure.

Potential mode of indirect transmission could either be iatrogenic or by flying insects. Iatrogenic transmission in the extent observed in this outbreak is very unlikely as needles were changed between groups. Therefore, if transmission would have occurred by vaccination it should have increased the transmission within the groups and not between the groups. Also, vaccine is administered subcutaneously and it was shown previously that this way of inoculation would mostly cause localized disease [8] while most of the animals affected through the described outbreak had generalized disease. Therefore, the most likely mode of transmission is by blood sucking flying insects. As mosquitoes are vessel feeders, injecting their saliva directly into the blood stream, they might be candidates for transmission. However, the mosquito female feeds on blood only before oviposition. The time period between blood feeding to oviposition is 2-5 days [19]. Adding this time to the assumed incubation period for LSDV would give a lag period of at least 9 days between mosquito feeding on a primary infected cow and appearance of clinical signs in a secondary infected cow (it is more likely that this time period is longer because this estimation assumes the second blood meal to take place at the same day of oviposition). Given the optimal lag period calculated from the model (around 7 days) transmission by mosquitoes through major blood meals seems unlikely. Failure to demonstrate virus persistence in *Culex *spp. for a long enough time [6] does not support this hypothesis either.

A more reasonable explanation is that virus is mechanically transmitted by interrupted feeding. This type of feeding is typical for the stable fly *Stomoxys calcitrans *[20], but the fact that it is a pool feeder [21] and the failure to demonstrate transmission by this fly [6] decrease the likelihood of this fly as being the primary vector of this virus. Another possibility is that a mosquito species that is an interrupted feeder may serve as the vector. Persistence of the virus in *Culex *mosquitoes and primarily in *C. pipiens *which is highly abundant in Israel [22], should be further investigated as well as feeding habits of these mosquitoes on cattle. In light of this study's findings, unravelling the mechanism of mechanical transmission of LSDV is of high interest and significance.

Mathematical modeling shows that the ratio of biting insect numbers to host numbers is positively correlated with the transmission probability [23]. It was demonstrated by Torr et al. that increasing the number of cattle heads in a herd increased the number of captured biting flies (including *Stomoxys *spp.) by a smaller factor, therefore reducing the vector to host ratio [24]. This indicates that for a vector-borne disease, increase in cattle density should decrease the probability of viral transmission. If direct contact would have played an important role in transmission, we would expect a positive correlation between cattle density and final attack rate, as higher density would cause higher contact rate [25]. We did not find an association between low density of cattle in a group and the risk of infection. Therefore this part of the analysis cannot support a higher significance for either direct or vector-borne indirect transmission.

Suckling calves showed the lowest attack rate, though in the dynamic model younger cattle did not show higher susceptibility to infection. We are not aware of previous reports of age related susceptibility to LSD. A possible alternative explanation for the lower attack rate in suckling calves may be associated with lower susceptibility of calves to biting by flies as previously described [24]. Another potential explanation can be associated with location, as the lowest attack rate was documented in the suckling calves which were located up to 100 meters from the other groups. Since the groups are homogenic, confounding between these potential causes can occur and cannot be fully accounted for.

A possible criticism of this study may be the lack of accurate surveillance during the initial phases of the outbreak. Unfortunately, this problem exists in many outbreaks. Frequently, there is a lag between initial appearance of clinical signs and the understanding that an outbreak is occurring. We overcame this problem by trying various scenarios (representing different sequences of case occurrence) extrapolated for the initial days of the outbreak from the data that we had. All scenarios gave the same results. The study can also be criticized by the fact that quite early in the course of this outbreak intervention was initiated, which included increased use of insecticides and vaccination of the herd, and most importantly immediate culling of the animals. It is improbable that vaccination with a live attenuated vaccine had any affect during the time frame of the outbreak, since the time for virus propagation and induction of immunity (when administered to sheep) takes one month [26], while most cases occurred within 10 days of vaccine administration. Furthermore, during later outbreaks of LSD, which occurred in 2007 in Israel it was shown that this vaccine had low efficacy [5]. Increased use of insecticides and more importantly early culling probably had a significant effect on outbreak spread and indeed were simulated in the model by inclusion of a constant *c *to differentiate cases that stayed in the herd from cases that were culled immediately. Since these interventions were imposed equally for all herd groups, the exact value of *c *should not have any effect on the final conclusion of this study.

It is possible that in this outbreak there were animals that were sub-clinically infected and were therefore not detected. In an experimental infection of calves by LSDV, Babiuk et al. showed that the highest levels of virus were detected in the skin nodules [9]. Their results support a significantly higher role of clinically affected animals (showing skin lesions) in virus transmission as compared to sub-clinically infected cattle. It can therefore be assumed that neglecting potential sub-clinically infected animals did not influence the calculation of transmission parameters. Abundance of sub-clinically infected or non-susceptible animals can also change outbreak dynamics by competing with susceptible animals on contact with infectious cattle. However, the sensitivity analysis that we performed showed that the abundance of such non-susceptible, possibly sub-clinically infected cattle, would not change the main conclusion of this study and would even support it (Figure 7b).

In conclusion, direct transmission would not support the development of an outbreak of LSD and control efforts should be mostly aimed at preventing indirect transmission. The lack of any other possible indirect transmission mechanism as well as previous experimental infections suggests that this route of transmission is mainly vector borne. Therefore, the control of the vector population may seem beneficial for preventing outbreak spread. The results regarding transmission through milking procedure may suggest that transmission by this route may be important. However, as we explained earlier, this result is not conclusive. Hence, until proven otherwise, milking of LSD sick cows should be stopped.

Finally, one should remember that in this outbreak all the animals showing severe disease were culled almost immediately when the generalized disease was observed, therefore significantly reducing the possibility for spread via water contaminated with saliva or nasal discharge and mutual grooming which might be increased during later stages of the disease. Further epidemiological investigation of LSDV in other settings is therefore warranted.

## Competing interests

The authors declare that they have no competing interests.

## Authors' contributions

All authors read and approved the final manuscript. RMC designed and constructed the model, performed statistical and sensitivity analysis and drafted the manuscript. YL designed and constructed the model, performed statistical and sensitivity analysis and drafted the manuscript. YH collected the data, performed initial laboratory diagnosis and performed the GIS work. EO and AA collected the data in the field and participated in outbreak investigation. ET helped in study conduction and laboratory diagnosis. NYS participated in outbreak investigation and study design. EK initiated the study, designed the study and the model, drafted the manuscript and performed the statistical analysis.

## Supplementary Material

Additional file 1 **Alternative fitting method for the Ein Zurim Lumpy skin disease data from 2006 - the inverted model**. Description of an alternative fitting method in which infection incidence is fitted to the prevalence of infection. Click here for file
