# Supplementary material for: Mathematical modelling and evaluation of the different routes of transmission of lumpy skin disease virus
Source: Vet Res. 2012 Jan 11;43(1):1. doi: 10.1186/1297-9716-43-1 (PMC3268087; doi:10.1186/1297-9716-43-1)
Supplement: Additional file 1 — Alternative fitting method for the Ein Zurim Lumpy skin disease data from 2006 - the inverted model. Description of an alternative fitting method in which infection incidence is fitted to the prevalence of infection. [file 1297-9716-43-1-S1.DOC]

**Additional file 1:**

**Alternative fitting method for the Ein Zurim Lumpy skin disease data from 2006 – the inverted model:**

We can estimate $z_{ij}$, the number of infected cows in group j in day i, based on the observation that they were sick a few days later: $z_{ij}=\sum_{i*>i} \frac{1}{\sqrt{2\pi}\sigma}e^{-\frac{\left(i-i^{*}-\mu\right)^{2}}{2*\sigma^{2}}}y_{i*j}$.

$z_{ij}$ should be related to the presence of sick cows in the different groups:

$$\frac{z_{ij}}{N_{ij}}=\alpha\sum_{k\in\text{all groups}} y_{ik}+\beta y_{ij}+\gamma\sum_{k\in\text{join milking groups}} y_{ik}+\delta\sum_{k\in\text{neighboring groups}} y_{ik}$$

where $y_{ik}$ is the number of sick cows in day i in group k. We can thus perform an optimization of :

$err=\sum_{i,j} \left[\frac{z_{ij}}{N_{ij}}-\alpha\sum_{k\in\text{all groups}} y_{ik}+\beta y_{ij}+\gamma\sum_{k\in\text{join milking groups}} y_{ik}+\delta\sum_{k\in\text{neighboring groups}} y_{ik}\right]^{2}$.

As can be seen by the sensitivity analysis (Figure S1), the results of this model are qualitatively similar to the results of the original model and the conclusion is even reinforced as the ratio (β + γ +δ)/α becomes even smaller. This approach has, however, one fundamental caveat; since the results are fitted by the number of cows that are assumed to be infectious, the fit should also include the early cases occurring until day 16. As we do not know the exact date of occurrence of these cases this fitting procedure may be problematic.


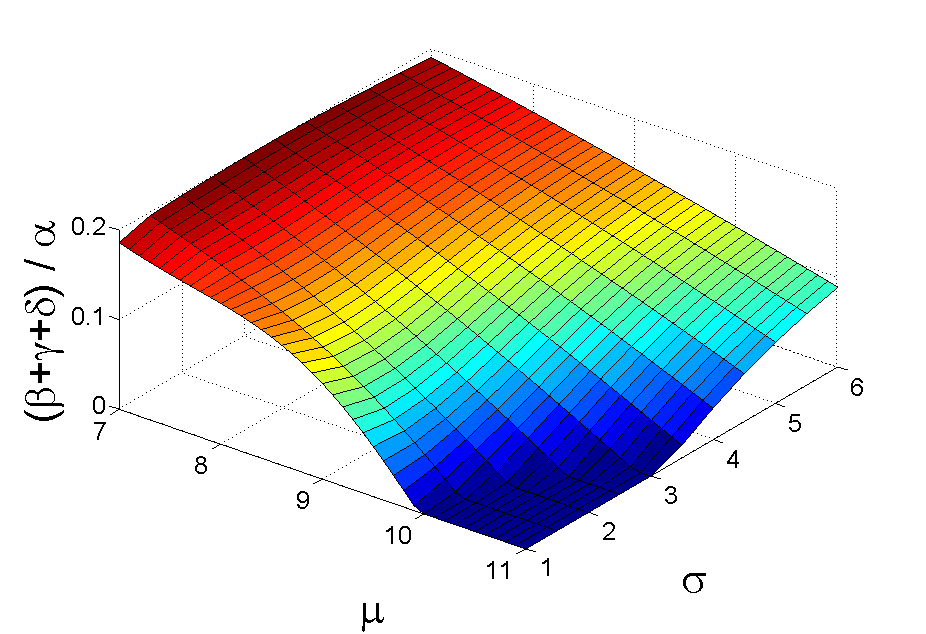


Figure S1: Sensitivity analysis of the ratio between direct (beta + gamma+ delta) and indirect (alpha) transmission rates as a function of the fixed average and variance of the incubation time. Parameters were estimated by the inverted fitting method.
